# Strengthening National Immunization Technical Advisory Groups in resource-limited settings: current and potential linkages with polio national certification committees

**DOI:** 10.1186/s12961-020-00632-7

**Published:** 2020-10-06

**Authors:** Sharon A. Greene, Blanche-Philomene Melanga Anya, Humayun Asghar, Irtaza A. Chaudhri, S. Deblina Datta, Morgane E. Donadel, Koffi Isidore Kouadio, Abigail M. Shefer, Kathleen F. Cavallaro

**Affiliations:** 1 Centers for Disease Control and Prevention, Atlanta, United States of America; 2 World Health Organization, Regional Office for Africa, Brazzaville, Republic of the Congo; 3 World Health Organization, Regional Office for the Eastern Mediterranean, Cairo, Egypt

**Keywords:** Advisory committee, Immunization programme, Vaccine decision-making, Health policy, Health workforce capacity-building, National Immunization Technical Advisory Group (or NITAG), Polio eradication, Polio endgame

## Abstract

**Background:**

Countries are transitioning assets and functions from polio eradication to integrated immunization and surveillance activities. We assessed the extent of linkages between and perceptions of National Immunization Technical Advisory Groups (NITAGs) and National Certification Committees (NCCs) for polio eradication to understand how linkages can be leveraged to improve efficiencies of these expert bodies.

**Methods:**

During May 2017 to May 2018, we administered a 15-question survey to a NITAG chair or member and an NCC counterpart in all countries of the WHO Regions for Africa (AFR) and for the Eastern Mediterranean (EMR) that had both a NITAG and an NCC. Data were analysed using frequency distributions.

**Results:**

Of countries with both a NITAG and an NCC (*n* = 44), the response rate was 92% (22/24) in AFR and 75% (15/20) in EMR. Some respondents reported being very familiar with the functions of the other technical bodies, 36% (8/22) for NITAG members and 38% (14/37) for NCC members. Over 85% (51/59) of respondents felt it was somewhat useful or very useful to strengthen ties between bodies. Nearly all respondents (98%, 58/59) felt that NCC expertise could inform measles and rubella elimination programmes.

**Conclusions:**

We observed a broad consensus that human resource assets of NCCs may serve an important technical role to support national immunization policy-making. At this stage of the polio eradication initiative, countries should consider how to integrate the technical expertise of NCC members to reinforce NITAGs and maintain the polio essential functions, beginning in countries that have been polio-free for several years.

## Background

The primary goal of national immunization programmes is to achieve high immunization coverage to prevent and control vaccine-preventable diseases, with the aim of meeting the Sustainable Development Goal for reducing childhood mortality [1]. This goal is complicated as the immunization landscape has become increasingly complex with the availability of new and under-utilised vaccines and delivery methods. The WHO has supported strengthening national decision-making processes and has emphasised the importance of establishing a National Immunization Technical Advisory Group (NITAG) to make evidence-based recommendations on immunization policy [2]. A NITAG should be comprised of 10–15 national experts who are independent of the government with the technical capacity to systematically evaluate vaccine introduction, priorities, schedules, target groups, immunization strategies, and safety issues to guide national policies and strategies based on local epidemiology and cost-effectiveness [2–4].

The Global Vaccine Action Plan 2011–2020 proposed that all countries establish or have access to a NITAG by 2020; good progress had been made –— as of 2017, nearly 70% of 194 WHO member states (subsequently referred to as countries) reported the establishment of a NITAG [5]. However, there is variation in the status of countries meeting the process indicators that define a well-functioning NITAG; globally only 98 (73%) of 134 countries with an existing NITAG meet the six process indicators [5, 6]. Research demonstrates that, for countries lacking a NITAG, constraints to establishing and sustaining this type of expert advisory body include a lack of human resources, insufficient skills on how to conduct an evidenced-based review and uneven recognition of the NITAG by the ministry of health [7–9]. Further, it is not clear to what extent NITAG recommendations lead to the implementation of immunization policy [9, 10]. In general, certain strategies are needed to help countries with constrained resources to integrate and strengthen NITAGs.

Another technical body whose function relates to immunization and provides independent technical advice at the country level is the National Certification Committee (NCC) for poliomyelitis eradication. The NCC includes national experts in scientific disciplines relevant to public health, including clinical medicine and virology. Between 1990 and 2003, NCCs were established in nearly every country to standardise the process of documentation and verification of progress toward polio eradication at the country level [11]. The NCC is responsible not only for providing a statement to the corresponding Regional Certification Commission, summarising the evidence on the country’s polio-free status, but also making recommendations to the national polio programme about risk mitigation and corrective actions [12].

As progress has been made toward global interruption of wild poliovirus transmission, the Global Polio Eradication Initiative (GPEI) partners have begun transitioning polio assets to concurrently sustain a polio-free world after eradication, while strengthening immunization systems to achieve other health priorities [13]. Even after global certification of wild poliovirus eradication, countries will need to maintain NCCs for poliovirus containment activities and to address circulating vaccine-derived polioviruses. In the long term, the expertise in polio eradication and methods for assessing country status are tangible assets that could strengthen policy-making for immunization services and for the control and elimination of other vaccine-preventable diseases. In the context of polio transition, we conducted a preliminary study to understand the extent of current linkages between the NITAGs and NCCs and how these existing linkages can be leveraged to improve country-led immunization policy development, while maintaining polio assets.

## Methods

The GPEI 2019–2023 polio eradication and endgame strategy calls for transition planning to ensure that polio assets and functions are integrated in existing public health initiatives [14]. Countries in the WHO Regions for Africa (AFR) and the Eastern Mediterranean (EMR) were selected for inclusion because countries in these regions not only have the most polio assets, including human resources, infrastructure and operational processes, but are also a high priority for transition planning [13]. A standardised 15-question (multiple choice and short answer) self-administered survey was developed to assess the range of technical expertise and scientific disciplines represented by NITAG and NCC members, the frequency of meetings or communications between the NITAG and NCC, perceptions on the value of committee service, and opinions on the role of the NCC after certification of global polio eradication. The English language survey instrument was pilot tested in Egypt with two immunization programme and policy experts. The final questionnaire was amended and translated into French for use where needed.

The presence of a NITAG in AFR and EMR countries in 2017 was identified from the WHO’s Annual Joint Reporting Form or via information received from the WHO Regional Officer for NITAGs^1^ [15]. All WHO member states in both AFR (*n* = 47) and EMR (*n* = 21) regions have an NCC, whereas the presence of a NITAG was reported in 24 (51%) countries in AFR and in 20 (95%) countries in EMR. An invitation to complete the paper survey was sent by email to the committee chairpersons of both NITAG and NCC, with the request to have one survey completed by each expert body in each country.

Scanned survey responses were collected during May 2017–2018 and entered into an electronic database. The frequency distribution for each variable of interest was used to summarise the data on attitudes, practices and perceived barriers. Responses were analysed by geographic region and expert body membership. There was a low proportion of item non-response; where data were missing, non-response was not included in the final proportion and thus the denominator for each proportion varied. Data were analysed in SAS version 9.4 (SAS Institute, Cary, NC, USA).

This project was determined to be non-research by the United States Center for Global Health Human Subjects Office, Centers for Disease Control and Prevention.

## Results

### Response rate

Overall, 40 countries of 44 eligible countries (91%) submitted a response from at least one expert committee member; 22 responses were received from NITAG members and 37 from NCC chairs or other members. Nineteen countries submitted a response from both a NITAG and an NCC member. In AFR, 50% (*n* = 8) of NITAG respondents and 59% (*n* = 13) of NCC respondents chaired the committee; in EMR, 83% (*n* = 5) of NITAG and 93% (*n* = 14) of NCC respondents chaired the committee. NITAG members were less likely to complete the survey than NCC members in both AFR (70% (*n* = 16) vs 92% (*n* = 22)) and EMR (30% (*n* = 6) vs. 75% (*n* = 15)), respectively, although the response rate was higher for both NITAG and NCC members in AFR than in EMR (Table 1).

**Table 1:** Survey response according to expert committee and WHO region, NITAG–NCC linkage survey, May 2017–May 2018

	African Region *N* = 47 *n* (%)	Eastern Mediterranean Region *N* = 21 *n* (%)
Survey response		
Number of countries with an NCC	47 (100%)	21 (100%)
Number of countries with an NCC and NITAG	24 (51%)	20 (95%)
Response by NCC among countries with both committees	22 (92%)	15 (75%)
Response by NITAG among countries with both committees	16 (70%)	6 (30%)

*NCC* National Certification Committee, *NITAG* National Immunization Technical Advisory Group

### Length of service

NCC survey respondents reported a median of 10 and 8 years of serving on the NCC in AFR (*n* = 22) and EMR (*n* = 15), respectively, with a range of 1–20 years. In contrast, the length of service reported by NITAG committee members in AFR (*n* = 16) and EMR (*n* = 6) varied by region; NITAG committee members reported a shorter term of service in AFR, with a median of 2.8 years (range 0–7) compared to 7.5 years (range 1–10) in EMR.

### Scientific expertise

**Fig. 1:**
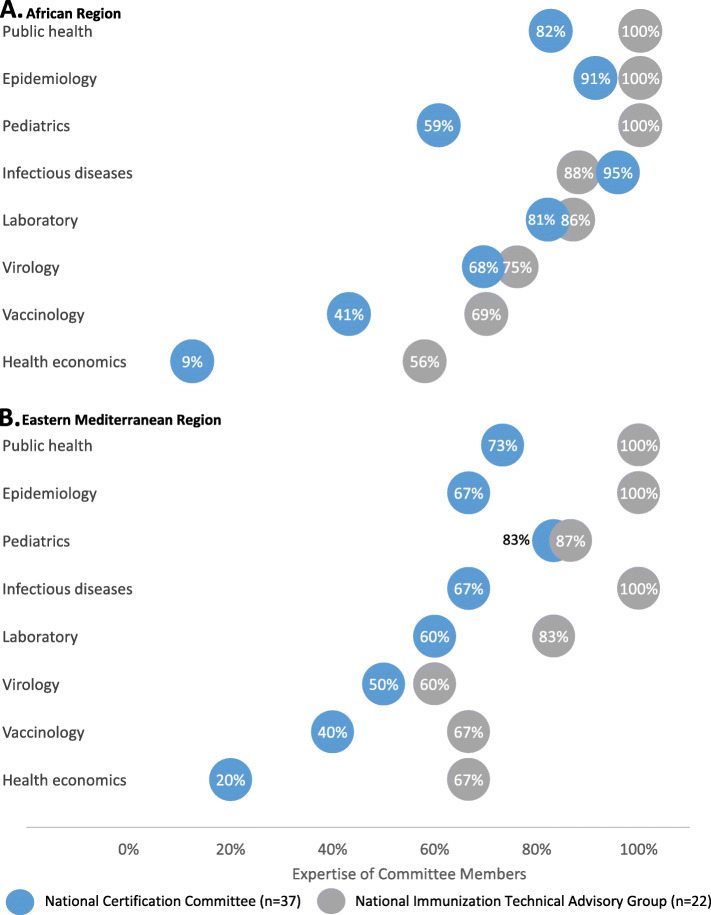
Reported expertise of NITAG and NCC members by WHO region. NITAG–NCC linkage survey, May 2017–May 2018. *NCC* National Certification Committee, *NITAG* National Immunization Technical Advisory Group

The NITAG and NCC committee members reported expertise in various scientific disciplines, including infectious diseases, clinical medicine and laboratory science. There was a high degree of overlap in expertise between the committees and by geographic region (Fig. 1). Over two-thirds of the respondents on both committees reported expertise in public health, epidemiology and paediatrics. Less than half of the NCC committee members reported expertise in vaccinology (15/37) or health economics (5/37) compared to over half of the NITAG committee members with expertise in vaccinology (15/22) and health economics (13/22). A higher proportion of the NITAG respondents reported expertise in all disciplines compared to the proportions reported by the NCC respondents in the same disciplines. Moreover, the NITAG members reported experience with health systems and delivery of immunization programmes in open-ended responses, whereas these experiences were not reported by any of the NCC members (data not shown).

### Common membership and linkages between the committees

The survey showed considerable membership overlap between the committees within a given country. Among the 18 countries with responses from both NITAG and NCC, 61% (11/18) of the NITAG and 50% (9/18) of the NCC respondents stated that there was shared membership, ranging from one to at least five members. The extent of shared membership across the committees was similar by region. When asked if any NCC members also serve on the NITAG in these 18 countries, 60% (9/15) and 67% (2/3) of NCC respondents in AFR and EMR, respectively, indicated shared membership.

**Fig. 2:**
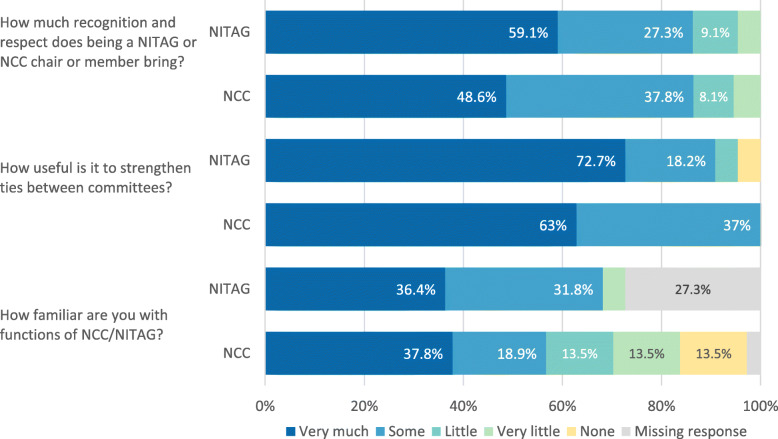
Perceptions on the value of committee membership and integration of committee functions, NITAG-NCC linkage survey, May 2017–May 2018. *NCC* National Certification Committee, *NITAG* National Immunization Technical Advisory Group

Less than half of the respondents in both regions reported being very familiar with the function of the other technical advisory group in their respective countries; 36% (8/22) of NITAG members were very familiar with the NCC functions and 38% (14/37) of NCC members were aware of NITAG functions (Fig. 2). Linkages between the two committees were explored by asking about regular communications with the other committee.

**Fig. 3:**
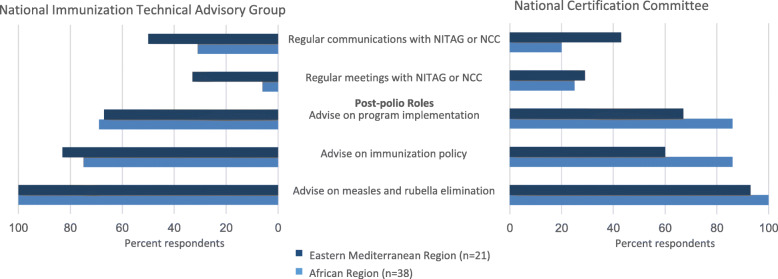
Existing linkages between NCC and NITAG committees and beliefs on how the technical assets of the NCC could benefit NITAG after polio eradication, NITAG–NCC linkage survey, May 2017–May 2018. *NCC* National Certification Committee, *NITAG* National Immunization Technical Advisory Group

Regular communications between the NITAG and NCC were reported less commonly in AFR, with 20% (4/20) of NCC respondents compared to 43% (6/14) in EMR (Fig. 3). The survey responses showed that countries generally do not have regular joint meetings between committees – both NITAG and NCC representatives from only one country in EMR reported regular joint meetings. Over 90% of all respondents reported that it would be somewhat or very useful to strengthen ties between the advisory committees (Fig. 2).

### Perceptions of committee membership and compensation

More than 85% of the NITAG (19/22) and the NCC (32/37) respondents reported receiving some to very much recognition and respect due to committee participation (Fig. 2). Respondents reported that over 80% (13/16) of the NITAG members and 95% (21/22) of the NCC members in AFR received monetary compensation, whereas 33% (2/6) of the NITAG and 13% (2/15) of the NCC members in EMR received monetary compensation. In the AFR, 50% (8/16) of the NITAG members specified that they received compensation through a sitting fee or honorarium, 56% (9/16) for travel and 25% (4/16) for both compared to 82% (18/22) of the NCC members receiving compensation through a sitting fee or honorarium, 41% (9/22) for travel and 33% (6/22) for both.

### Role of NCC technical expertise after polio eradication

Respondents were asked several questions on the potential for the NCC members to serve in immunization advisory roles after wild poliovirus eradication when the need for regular NCC meetings and review of polio surveillance activities is likely to decrease. Nearly all of the NITAG and NCC members affirmed that NCC expertise would be an important post-polio role for other vaccine-preventable disease programmes, including advice on measles and rubella elimination (Fig. 3). At least three-quarters (17/22) of the NITAG members indicated that NCC’s technical expertise would be a good fit for immunization policy and 68% (15/22) of NITAG members felt that NCC members should contribute to immunization programme implementation. Finally, 86% (19/22) of the NCC respondents in AFR felt that their skills would be equally useful for immunization policy and programme implementation, whereas less than 60% (9/15) of the NCC respondents in EMR agreed.

## Discussion

Given the number of years that some countries in EMR and AFR have been free of poliovirus circulation, the process of transitioning assets and functions from dedicated polio eradication activities to other global health priorities is underway. As an annual function, NCCs have the responsibility to assess population immunity and poliovirus surveillance data for the risks of poliovirus transmission and the ability to promptly detect and respond to wild poliovirus importation, vaccine-derived poliovirus circulation or poliovirus release from containment. In this regard, it is logical to consider the most effective ways to not only maintain NCC expertise in these GPEI activities but also to apply these assets to aid national immunization decision-making and policy-making. We found that most responding committee members on NITAGs and NCCs in both regions were not fully knowledgeable of the functions of the other technical body in their countries. While some respondents did report cross-membership, it was uncommon for the NITAG and NCC bodies to regularly meet or communicate. There was a broad consensus that the NCC members could contribute toward the verification of measles and rubella elimination and that strengthening ties between the NITAG and NCC would be useful for informing immunization policy.

Through the endorsement of the Global Vaccine Action Plan at the World Health Assembly in 2012, all countries committed to establishing a functional NITAG by 2020; subsequent global discussion has further refined this goal, noting that all countries should have access to a designated NITAG [8]. Efforts to expand and strengthen NITAGs revealed that one key to success is institutional integration because this enhances the acceptance of expert review processes and ensures structural and functional sustainability [10]. Our survey showed that less than 50% of the NITAGs and NCCs have regular communications and that only one country reported joint meetings. Strong links to other in-country technical bodies may enhance the extent to which a NITAG becomes embedded in the flow of evidence into policy because the reputation, capacity and quality of work by the committee facilitates the uptake of health policies by decision-makers [16]. Efforts to enhance consultations and include NCC members into NITAG activities may broaden the NITAG membership pool and strengthen evidence-based processes.

Analysis of WHO data reveals that challenges to the establishment of NITAGs include identifying adequate expertise and undertaking quality review [17]. This survey indicated that there is substantial overlap in member expertise between NITAGs and NCCs. The NCC’s role is to review national programmatic evidence on poliovirus vaccine coverage and surveillance indicators and then make recommendations to strengthen the core programmatic functions. This aligns well with one of NITAG’s roles in making evidence-based recommendations to strengthen the immunization programmes in addition to introduction of new and under-utilised vaccines. One important distinction is that NCC members have reviewed polio programme indicators and provided detailed annual statements on the country’s polio progress for nearly a decade, on average, whereas NITAGs are a more recent expert body serving to enhance national immunization programmes and their members in our survey reported less than 4 years, on average, advising national health authorities. This suggests that there is opportunity for NCC members to assist NITAGs in gaining a fundamental understanding of the processes for making recommendations based on subnational surveillance and immunization coverage data to bolster immunization programmes.

It is recommended that the NITAG members have a diverse range of skills and expertise [2]. While there was a broad overlap between the NCC and NITAG members with regards to expertise areas, there was a small proportion of NCC members reporting expertise in health economics. Economic analysis is an integral factor in formulating policy decisions and identifying the most efficient vaccination strategies at the country level. A consensus framework developed by experts on modelling and immunization decision-making recommended that health economic evaluations of vaccines be considered by NITAGs, particularly when considering the introduction of a new vaccine [18].

Key informant interviews with the NITAG members in low- and middle-income countries in a recent study highlighted that conducting budget impact analyses to facilitate decision-making is a technical challenge for NITAGs [19, 20]. Linkages between the bodies could be encouraged by establishing a liaison membership for the NCC to the NITAG or providing an opportunity for the NCC to report to the NITAG on their annual review and recommendations. Creating opportunities for communication, collaboration and awareness of each body’s scientific expertise may help not only to improve the functional capacity of NITAGs but may provide an alternative source of experts when additional support is needed.

### Limitations

Our study has several limitations. First, we only interviewed the committee members from countries with a NITAG in AFR and EMR, which is not be generalizable to other regions or to all countries in those regions trying to establish a technical advisory body. Second, the presence of a NITAG was identified from the WHO’s Annual Joint Reporting Form (JRF) or via information received from the WHO Regional Officer in charge of routine immunization and NITAGs. The Joint Reporting Form is a self-reported data source that was not verified against other records and we did not evaluate whether the NITAG met the process indicators for a functional committee. Third, because more NCC members responded to the survey, the reported linkages and perceptions may be weighted toward the NCC perspective. Fourth, we did not inquire whether there were differences between countries and committees on duration of term appointments. Lastly, findings were based on the participants’ perceptions and may not be representative of all members of those bodies.

## Conclusions

This survey provided baseline information that can help guide country-level stakeholders to strengthen NITAGs; establishing a framework allowing support from experienced NCC members to assess programmatic data may better inform decision-making on immunization programme policy. NCC member expertise may be particularly useful in supporting the review of the quality of measles and rubella surveillance indicators, including participation on National Verification Committees for measles and rubella elimination in documenting progress toward elimination goals. Considering the similar expertise among members and related technical body functions, better integration of these and other in-country expert groups would enable countries to ensure a greater efficiency and sustainability of NITAGs.

## Data Availability

The datasets used and/or analysed during the current study are available from the corresponding author on reasonable request.

## Abbreviations

AFR: WHO Regional Office for Africa
EMR: World Health Organization Regional Office for the Eastern Mediterranean
GPEI: Global Polio Eradication Initiative
NCC: National Certification Committee for poliomyelitis eradication
NITAG: National Immunization Technical Advisory Group

## Footnotes

1 The 24 countries in AFR that have a NITAG are Algeria, Angola, Benin, Burkina Faso, Cameroon, Côte d’Ivoire, Democratic Republic of the Congo, Ethiopia, Kenya, Malawi, Mali, Mauritania, Mozambique, Niger, Nigeria, Senegal, South Africa, South Sudan, Swaziland, Togo, Uganda, United Republic of Tanzania, Zambia and Zimbabwe. The 20 countries in EMR that have a NITAG are Afghanistan, Bahrain, Djibouti, Egypt, Iran, Iraq, Jordan, Kuwait, Lebanon, Libya, Morocco, Oman, Pakistan, Qatar, Saudi Arabia, Sudan, Syrian Arab Republic, Tunisia, United Arab Emirates and Yemen.
